# HAPPi Kneecaps! A double-blind, randomised, parallel group superiority trial investigating the effects of sHoe inserts for adolescents with patellofemoral PaIn: phase II feasibility study

**DOI:** 10.1186/s13047-021-00498-0

**Published:** 2021-12-10

**Authors:** Isobel C. O’Sullivan, Kay M. Crossley, Steven J. Kamper, Marienke van Middelkoop, Bill Vicenzino, Melinda M. Franettovich Smith, Hylton B. Menz, Anne J. Smith, Kylie Tucker, Karina T. O’Leary, Nathalia Costa, Natalie J. Collins

**Affiliations:** 1 School of Health and Rehabilitation Sciences: Physiotherapy, The University of Queensland, Brisbane, Australia; 2 La Trobe Sport and Exercise Medicine Research Centre, School of Allied Health, Human Services and Sport, La Trobe University, Melbourne, Australia; 3 Faculty of Medicine and Health, The University of Sydney, Sydney, Australia; 4 Nepean Blue Mountains Local Health District, Penrith, Australia; 5 Department of General Practice, Erasmus MC Medical University Center, Rotterdam, The Netherlands; 6 School of Allied Health, Human Services and Sport, La Trobe University, Melbourne, Australia; 7 School of Allied Health, Curtin University, Perth, Australia; 8 School of Biomedical Sciences, The University of Queensland, Brisbane, Australia; 9 Surgical Treatment and Rehabilitation Services, Metro North Hospital and Health Service, Herston, Australia; 10 Faculty of Health and Behavioural Sciences, The University of Queensland, Brisbane, Australia

**Keywords:** Patellofemoral pain, Adolescents, Foot orthoses, Physiotherapy, Feasibility

## Abstract

**Background:**

Patellofemoral pain (PFP) affects one-third of adolescents and can persist into adulthood, negatively impacting health and quality of life. Foot orthoses are a recommended treatment for adults with PFP, but have not been evaluated in adolescents. The primary objective was to determine the feasibility of conducting a full-scale randomised controlled trial (RCT) evaluating effects of contoured, prefabricated foot orthoses on knee pain severity and patient-perceived global change, compared to flat insoles. The secondary objective was to describe outcomes on a range of patient-reported outcome measures.

**Methods:**

We recruited adolescents aged 12–18 years with PFP of ≥2 months duration into a double-blind, randomised, parallel-group feasibility trial. Participants were randomised to receive prefabricated contoured foot orthoses or flat shoe insoles, and followed for 3 months. Participants and outcome assessors were blinded to group allocation. Primary outcomes were feasibility of a full-scale RCT (number of eligible/enrolled volunteers; recruitment rate; adherence with the intervention and logbook completion; adverse effects; success of blinding; drop-out rate), and credibility and expectancy of interventions. Secondary outcomes were patient-reported measures of pain, symptoms, function, quality of life, global rating of change, patient acceptable symptom state, and use of co-interventions.

**Results:**

36 out of 279 (12.9%) volunteers (27 female, mean (SD) age 15 (2) years, body mass 60 (13) kg) were eligible and enrolled, at a recruitment rate of 1.2 participants/week. 17 participants were randomised to receive foot orthoses, and 19 to flat insoles. 15 participants returned logbooks; 7/15 (47%) adhered to the intervention. No serious adverse events were reported. 28% (10/36, 4 pandemic-related) of participants dropped out before 3 months. Blinding was successful. Both groups found the inserts to be credible.

**Conclusions:**

Based on a priori criteria for feasibility, findings suggest that a full-scale RCT comparing contoured foot orthoses to flat insoles in adolescents with PFP would not be feasible using the current protocol. Prior to conducting a full-scale RCT, feasibility issues should be addressed, with protocol modifications to facilitate participant retention, logbook completion and shoe insert wear.

**Trial registration:**

Australian New Zealand Clinical Trials Registry (ANZCTR): ACTRN12619000957190. Date registered: 8/07/2019.

## Background

Patellofemoral pain (PFP) is a common musculoskeletal condition affecting 30% of adolescents [1]. Pain is often aggravated by activities involving weightbearing on a flexed knee; for example, running, jumping, ascending and descending stairs, and squatting. Pain and disability associated with PFP in adolescents can affect participation in physical activity and sport, general and mental health, and quality of life [2–6]. PFP is persistent, even in adolescents, with 21% of 12- to 35-year-olds still reporting pain 6 years after an initial appointment with a general practitioner [7].

There are few evidence-informed recommendations to treat PFP in adolescents and thus, interventions developed for use in adults tend to be used in adolescents, often with poorer outcomes [8, 9]. Key differences between adolescents and adults may underpin worse outcomes in adolescents. Specifically, adolescents with greater hip abduction strength have an increased risk of PFP, while there is no association in adults [10]. Adults tend to present with reduced quadriceps strength [10], while adolescents demonstrate no quadriceps deficit until 16–18 years of age [4]. The presence of bilateral symptoms also differs between groups, with 55–60% of adults reporting bilateral symptoms [11, 12] compared to 70–79% of adolescents [13]. Poorer adherence to recommended treatments, particularly exercise therapy, also likely contributes to worse outcomes in adolescents [9]. Only two randomised controlled trials (RCTs) have investigated treatments specifically for adolescents with PFP [13, 14]. Their findings indicate that exercise therapy improves outcomes in this group, with further improvements arising when soft foot orthoses were prescribed [13, 14].

Foot orthoses are inserts worn in shoes that are contoured to the shape of the foot, and were recommended to treat PFP in the 2018 consensus statement [8]. Our previous work demonstrated the effectiveness of foot orthoses in adults with PFP [11, 15]. Compared to participants allocated to flat insoles or wait and see, adults with PFP who received prefabricated foot orthoses demonstrated greater global improvement at 6 weeks [11, 15], and a faster improvement in symptom severity [11]. This is important because duration and severity of symptoms predict poor long-term prognosis for PFP [16]. Although important preliminary information about the effects of foot orthoses in 10 female adolescents with PFP was provided in a pilot study [14], a modern approach to foot orthoses prescription with a larger, more diverse adolescent population is required. To inform the conduct of a full-scale RCT evaluating foot orthoses for adolescents with PFP, a feasibility trial is needed to determine whether contemporary clinical trial methods and foot orthoses prescription are feasible in this population.

The primary objective of this study was to determine the feasibility of conducting a full-scale RCT evaluating the effects of contoured, prefabricated foot orthoses on knee pain severity and patient-perceived global change in adolescents with PFP, compared to flat insoles. The secondary objective was to describe outcomes on a range of patient-reported outcome measures.

## Methods

### Experimental design

The HAPPi Kneecaps! Study (sHoe inserts for Adolescents with Patellofemoral PaIn) was a randomised, controlled, participant- and assessor-blind feasibility trial with two parallel groups (1:1 allocation ratio). Detailed study methods are available in the protocol paper [17]. We consulted the SPIRIT 2013 statement [18] and the CONSORT 2010 statement extension to randomised pilot and feasibility trials [19] in designing and reporting the study. Guidelines for trials modified due to COVID-19 [20] were also consulted. The University of Queensland Human Research Ethics Committee granted ethics approval (approval number 2018000159). The trial was prospectively registered on the Australia New Zealand Clinical Trials Registry on 08/07/2019 (ACTRN12619000957190).

### Participants

Adolescent volunteers were recruited from the community in Brisbane and the Gold Coast, Queensland, Australia. Several methods were used for recruitment, including advertising at community and school sporting events, and on websites and social media pages (Facebook, Instagram). Participants were also recruited from our existing database of PFP volunteers.

The inclusion criteria were: (i) aged 12–18 years; (ii) non-traumatic onset of anterior pain rated at least 3 on an 11-point numerical rating scale (0 = no pain, 10 = worst pain imaginable); (iii) pain aggravated by PFJ-loading activities (e.g., squatting, stair ambulation, running, jumping); and (iv) pain present for two months or more, and at some time during most weeks. Exclusion criteria were: (i) concomitant pain at sites other than the anterior knee (e.g. other knee structures, hip, lumbar spine); (ii) history of surgery on the ipsilateral limb or lumbar spine, or other suspected knee joint pathology (e.g. Osgood Schlatter’s Disease); (iii) planned lower limb surgery; (iv) recent PFP treatment (e.g. physiotherapy or knee joint injections in the last 3 months; foot orthoses prescription in the last 12 months); or (v) any foot condition impeding the prescription of foot orthoses.

All participants were required to provide written informed consent prior to participation. For participants under 18 years of age, their parent/guardian was also required to give written informed consent.

### Sample size

Formal sample size calculations were not conducted [21, 22]. We estimated that 40 participants (20 participants per group) would allow us to observe practicalities of recruitment, acceptability of the shoe inserts, adverse events, dropouts, and sample variability.

### Procedure

Eligibility was determined by one investigator (ICO, registered Physiotherapist) through two phases: (i) online screening (Research Electronic Data Capture [REDCap], Vanderbilt University, Nashville, USA); and (ii) physical screening at The University of Queensland to confirm the clinical diagnosis of PFP. Participants (and their parent/guardian if aged under 18 years) gave written consent and, after baseline measures were obtained, were randomised to receive either contoured foot orthoses or flat shoe inserts. We used simple randomisation, with the randomisation sequence generated by one investigator (KT) using a random number generator. The research assistant (KTO) held the randomisation sequence off-site to ensure concealed allocation, and was responsible for communicating group allocation to the physiotherapists. Study physiotherapists were unable to be blinded to group allocation due to the nature of the intervention. The primary investigator (ICO) was blinded to group allocation throughout the trial and collected primary outcomes. Secondary outcomes were self-reported by participants. Blinding of participants was maintained through communicating to each participant that there were two interventions that were being investigated, however, the two types of interventions and study hypotheses were not disclosed [11, 23].

### Interventions

Participants attended physiotherapy appointments at one of six clinics across Brisbane and the Gold Coast for fitting of their allocated shoe insert. Study physiotherapists were trained in fitting procedures, as used in previous studies to maximise comfort [23]. Each participant received up to four pairs of inserts, fit to shoes that would accommodate the inserts and provide support. Participants were asked to attend up to three appointments to allow for any adjustments to be made and ensure inserts were comfortable. Each person was asked to wear the inserts as much as possible throughout the week. Participants in both groups received a handbook, which provided general information and advice about PFP and activity [17].

#### Prefabricated contoured foot orthoses

Participants assigned to the contoured foot orthoses group received prefabricated foot orthoses (Vasyli Medical, Labrador, Australia). The orthoses are manufactured from ethylene-vinyl acetate (EVA) with options for a high (hard, Shore A 70°), medium (Shore A 55°) and low (soft, Shore A 45°) density orthosis with inbuilt medial arch support and varus wedging. Density was selected based on comfort, and modifications were made to the orthoses in the form of heat moulding or wedges to achieve a comfortable fit [17], based on our published algorithm [23]﻿.

#### Flat shoe insoles

Participants assigned to this group received flat shoe insoles made of the same high-density EVA as the contoured foot orthoses, which were a uniform thickness of 3 mm along their length. To facilitate blinding regarding the true intervention, the contoured foot orthoses and flat insoles were covered in the same fabric and markings. Participants randomised to this group were advised that the intervention enhanced sensory feedback, and the insoles were heat moulded as necessary to enhance comfort [17].

### Outcome assessment

At baseline, participant details were recorded, including demographics, knee/s affected, symptom duration and aggravating activities. Patient-reported outcome measures were collected at baseline, 6 weeks, and 3 months post-randomisation via an online platform (REDCap). For the duration of the study, participants were asked to complete a paper logbook to record details of daily activities, pain, and footwear [17]﻿. Study physiotherapists recorded attendance, prescription notes and adverse events during fitting and follow-up appointments. 3 months was nominated as the primary endpoint.

The primary outcome was the feasibility of conducting a full-scale RCT. Feasibility was assessed by evaluating the following outcomes:

(i) Number of eligible volunteers (from recruitment database).
(ii) Willingness of participants to enrol in the study (from recruitment database).
(iii) Recruitment rate (from recruitment database).
(iv) Adherence with allocated intervention and logbook completion (from participant logbook).
(v) Adverse events (from Study Practitioner notes, adverse events database, and participant logbook).
(vi) Success of blinding (risk of performance and detection bias, from the Credibility and Expectancy Questionnaire) [24].
(vii) Drop-out rate (from trial database; defined as participants who did not complete 3-month outcome measures).

Participants completed the Credibility and Expectancy Questionnaire [24] at baseline and at their second visit with the study physiotherapist (~ 2 weeks post-randomisation). The Credibility and Expectancy Questionnaire consists of six items in two sections; four items related to thoughts, and two items related to feelings. Credibility was derived from the first three thought items, and expectancy was derived from the fourth thought question and the two feeling questions [25]. The Credibility and Expectancy Questionnaire has been used in previous adolescent studies [26, 27].

Prior to the study, we set three criteria to determine the feasibility of a full-scale RCT: (i) a recruitment rate of 1 participant per week; (ii) adherence of at least 2 h of shoe insert wear per day for 5 days a week; and (iii) ≤20% drop out rate at 3 months [17].

The following secondary outcomes were collected by online questionnaires and participant logbooks (details provided in the protocol paper) [17]:

(i) Usual and worst knee pain severity during a self-nominated aggravating activity, measured using a 100 mm visual analogue scale (0 mm = no pain and 100 mm = worst pain imaginable) [28].
(ii) Knee injury and Osteoarthritis Outcome Score Child Version (KOOS-Child) [29].
(iii) Knee injury and Osteoarthritis Outcome Score – Patellofemoral Subscale (KOOS-PF) [30].
(iv) Global Rating of Change (GROC), using a 7-point Likert scale [13].
(v) Patient Acceptable Symptom State (PASS) [31].
(vi) Anterior Knee Pain Scale [32].
(vii) EuroQOL-5D-5L. The UK time-trade-off scoring algorithm was used to weight each participant’s profile data to produce a single EQ-5D index score (as there are no data published in Australian adult or adolescent populations) [33].
(viii) Use of co-interventions (e.g., pain medication, physiotherapy, knee brace, other footwear interventions).

### Statistical analyses

Analyses were conducted by a blinded investigator (ICO) using SPSS software (IBM SPSS Statistics for Macintosh, Version 27.0, IBM Corp., Armonk, NY, USA). Baseline data were checked for normality using Shapiro-Wilk tests and presented as mean and standard deviation (normal distribution) or median and interquartile range (not normal distribution). Primary feasibility outcomes for this study were presented using descriptive statistics. Descriptive statistics were calculated for secondary outcomes at all timepoints, separately for each group.

### Protocol deviations

There are two instances where our methods deviated from those reported in our protocol paper [17]. We were not able to report data from the Youth Quality of Life Short Form at any of the measured timepoints. This was due to an error with the online data collection platform. We also planned to provide a range of plausible estimates of treatment effects for prefabricated foot orthoses, compared to flat insoles, by reporting between-group differences (with 95% confidence intervals) for secondary outcome measures. Due to experiencing greater loss to follow-up than anticipated (including the impact of COVID-19), and the likely imprecision of between-group estimates from a small pilot trial [34], we elected not to report this. Instead, we only present descriptive statistics for secondary outcomes at each timepoint.

## Results

**Fig. 1:**
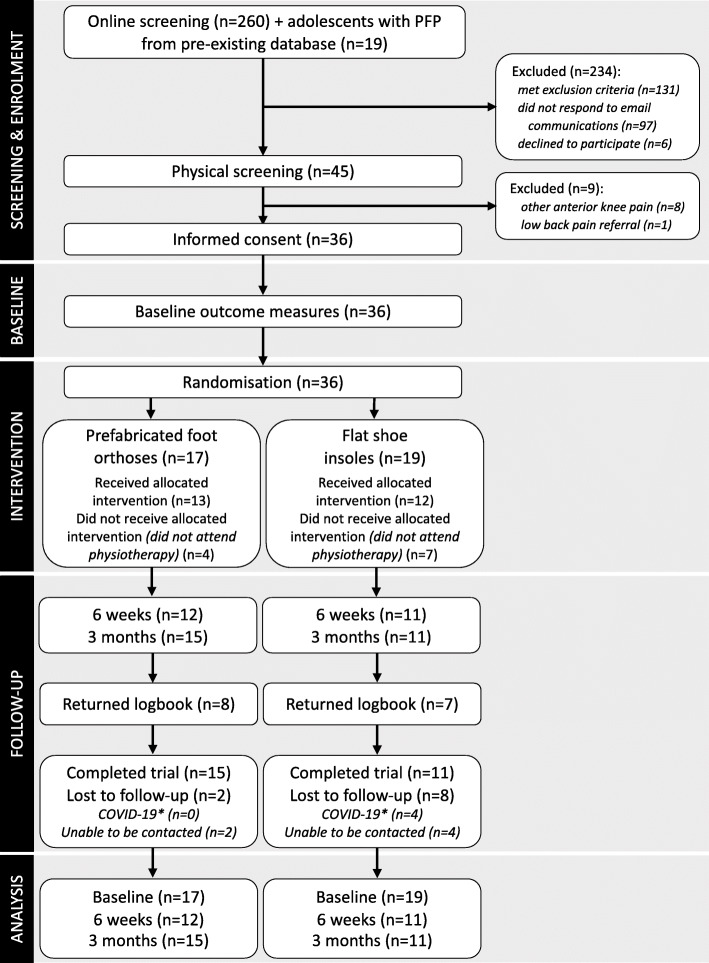
Participant flow through the study. *COVID-19 loss to follow-up refers to those whose participation in the trial was affected by COVID-19 restrictions

Between August 2019 and March 2020, 260 volunteers responded to advertisements and completed online screening (Fig. 1). An additional 19 volunteers were recruited from a pre-existing database. 149 volunteers were potentially eligible after online screening. Of these, 97 did not respond to email or telephone communications and 6 declined to participate in the physical screening. 45 consented to physical screening, from which 36 were eligible and enrolled in the study. The most common reasons for ineligibility via online screening were current foot orthoses use (*n* = 70, 53%), previous lower limb injury or surgery (*n* = 29, 22%) and other sources of anterior knee pain (*n* = 18, 14%). The most common reason for exclusion after physical screening was the presence of other sources of anterior knee pain (Osgood Schlatter’s Disease, *n* = 6; patellar tendinopathy, *n* = 2). In March 2020, recruitment was ceased at 36 participants due to COVID-19 restrictions.

Of the 36 participants randomised, 17 participants were allocated to receive contoured foot orthoses and 19 participants were allocated to flat insoles. 13 (76%) participants from the contoured foot orthoses group and 12 (63%) participants from the flat insole group attended appointments with their physiotherapist and were fitted with their allocated intervention. The mean number of appointments attended was 2.5 (SD 0.6) (contoured foot orthoses group 2.6 (0.7); flat insole group 2.4 (0.5)), and the mean number of inserts fitted was 2.6 (0.8) (contoured foot orthoses group 2.7 (1); flat insole group 2.6 (0.7)).  The remaining 11 participants did not attend physiotherapy appointments, and did not receive their allocated intervention. 3-month outcome measures were completed by 15 (88%) participants from the contoured foot orthoses group and 11 (58%) participants from the flat insole group (72% of total cohort). Participant characteristics are presented in Table 1.

**Table 1:** Baseline characteristics of participants. Values are mean (SD) unless otherwise indicated

Characteristics	Contoured foot orthoses (***n*** = 17)	Flat shoe insoles (***n*** = 19)	Total (***N*** = 36)
Age (years), median [IQR]	14 [5]	17 [3]	15 [4]
Number (%) of females	12 (71)	15 (79)	27 (75)
Height (cm)	165 (9)	169 (12)	167 (10)
Body mass (kg)	58.5 (12.1)	60.8 (14.4)	59.7 (13.3)
Number (%) with bilateral knee pain	11 (65)	10 (53)	21 (58)
Duration of knee pain months, median [IQR]	9 [38]	24 [18]	18.5 [29.5]
Average pain severity^^^, median [IQR]	50 [24]	50 [40]	50 [34]
Worst pain severity^^^, median [IQR]	70 [32]	70 [37]	70 [21]
Most painful activity in the last week, count (%):			
squatting	5 (29)	6 (32)	11 (30.5)
running	6 (35)	5 (26)	11 (30.5)
walking upstairs	4 (24)	4 (21)	8 (22)
walking downstairs	2 (12)	1 (5)	3 (8)
walking	0 (0)	2 (11)	2 (6)
jumping	0 (0)	1 (5)	1 (3)

^^^Measured on a 100 mm visual analogue scale (0 = no pain, 100 mm = worst pain imaginable), during their nominated most aggravating activity in the past week

*IQR* interquartile range

### Feasibility results (primary outcomes)

#### Number of eligible volunteers, willingness to enrol, and recruitment rate

12.9% (36/279) of all volunteers (including those who responded to advertisements and those who were recruited from a pre-existing database) were eligible and willing to enrol in the study. Of the 45 volunteers who underwent physical screening, 36 (80%) were eligible and provided informed consent to enrol in the study. Recruitment rate was 1.2 participants per week over 30 weeks.

#### Adherence

Fifteen of the 36 (42%) participants completed and returned the paper logbooks; 8 out of 17 from the contoured foot orthoses group, and 7 out of 19 from the flat insole group (Fig. 1). From the logbooks, 7 out of 15 (47%) participants met predefined minimum adherence with shoe insert wear (2 h per day, 5 days per week) for at least 7 out of 12 weeks; 5 participants from the contoured foot orthoses group, and 2 participants from the flat insole group. The logbook data showed that participants wore their allocated shoe inserts for a mean of 4.5 (SD 1.8) hours per day (contoured foot orthoses: 4.5 (1.5) hours per day; flat insoles: 4.4 (2.1) hours per day).

#### Adverse events

Only minor, transient adverse events were reported, predominantly in logbooks. In the contoured foot orthoses group, one participant reported one day of foot arch pain (week 3), and one participant reported experiencing blisters (weeks 1, 5 and 6) and one day of foot pain (week 2). In the flat insole group, two participants reported one day of blisters (week 1 and 2), and one participant reported foot pain on the first day of wear (week 1). One participant who received flat insoles contacted the investigators about the inserts rubbing in the second week of wear, causing minor discomfort and skin redness. This resolved in two days, and the participant was able to continue wearing the inserts. .

#### Success of blinding

**Fig. 2:**
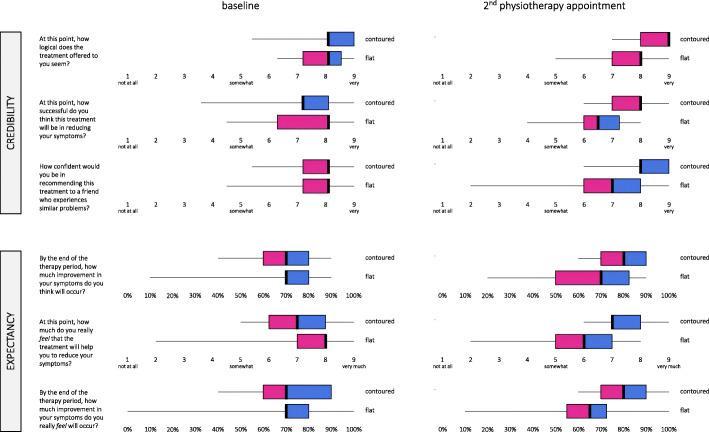
Credibility and Expectancy Questionnaire data at baseline and 2nd physiotherapy appointment. Left whisker: quartile 1; pink box: quartile 2; black line: median; blue box: quartile 3; right whisker: quartile 4

Figure 2 presents data from the Credibility and Expectancy Questionnaire. There were no notable differences in median scores between groups at baseline or after being fitted with their allocated shoe inserts.

#### Drop-out rate

Dropout rate was 28% (10/36) at 3 months.

### Secondary outcomes

Table 2 presents secondary patient-reported outcome measures at baseline, 6 weeks, and 3 months. Of the 15 participants who returned logbooks, 3 participants reported using co-interventions for their knee pain. One participant in the contoured foot orthoses group used paracetamol (1 occasion). In the flat insole group, one participant used paracetamol (3 occasions) and ice (1 occasion), and one participant used ice (15 occasions).

**Table 2:** Secondary outcome measures at baseline, 6 weeks and 3 months (mean (SD) unless otherwise stated)

	Contoured foot orthoses (***n*** = 17)			Flat shoe insoles (***n*** = 19)		
	Baseline	6 weeks^**a**^	3 months^**b**^	Baseline	6 weeks^**c**^	3 months^**d**^
Average pain severity [0-100 mm]	47.7 (16.1)	46.1 (21.7)	38.1 (21.2)	47.5 (23.2)	40.6 (23.5)	33.4 (20.7)
Worst pain severity [0-100 mm]	63.8 (16.7)	60 (23)	47.9 (23.8)	57.9 (24.4)	51.8 (28.8)	42.7 (24.1)
GROC, *number (%)*						
*completely recovered*	–	0 (0)	1 (6.5)	–	0 (0)	0 (0)
*strongly recovered*	–	4 (33)	8 (53.5)	–	3 (27.3)	4 (36)
*slightly recovered*	–	5 (42)	4 (27)	–	3 (27.3)	5 (46)
*same*	–	2 (17)	1 (6.5)	–	3 (27.3)	2 (18)
*slightly worse*	–	0 (0)	0 (0)	–	1 (9)	0 (0)
*much worse*	–	1 (8)	1 (6.5)	–	1 (9)	0 (0)
Number (%) responding ‘yes’ to Patient Acceptable Symptom State	12 (71)	9 (75)	13 (87)	13 (68)	7 (64)	8 (73)
KOOS-Child [100–0]						
*Pain*	63.4 (10.6)	66.7 (14.4)	81.3 (12.3)	63.5 (16.8)	68.2 (19.1)	79 (13.7)
*Symptoms*	77.5 (9.9)	77.1 (13.4)	88.5 (10.1)	75.4 (13.2)	77.6 (12.7)	84.7 (12.1)
*ADL*	82.2 (9.5)	86.4 (10.6)	94 (6.5)	79.1 (14.5)	87.2 (9.7)	93.8 (7)
*Sport and play*	62.4 (16.5)	69.3 (23.4)	81.6 (17.4)	62.2 (20.3)	72.1 (25.4)	87.3 (16.3)
*QoL*	57.1 (17.1)	61.1 (20.6)	78 (17.8)	52.9 (18.5)	64 (20)	75.4 (18.5)
KOOS-PF [100–0]	60.6 (15.8)	65.1 (23)	82.6 (13.7)	58 (16.9)	70.9 (17.4)	80.4 (19.6)
Anterior Knee Pain Scale [100–0]	74.7 (10.7)	75.5 (13.6)	85.5 (10.2)	72.6 (10.8)	79.4 (13.3)	87.5 (9.8)
EQ-5D-5L index value [1–0]	0.72 (0.1)	0.68 (0.28)	0.83 (0.13)	0.71 (0.19)	0.76 (0.13)	0.85 (0.12)
EQ-5D-5L VAS [100–0]	76.9 (15.1)	81.6 (20.7)	80.2 (17.7)	81.8 (10.7)	83 (12.6)	88.4 (7.6)

*GROC* global rating of change, *KOOS* Knee injury and Osteoarthritis Outcome Score, *ADL* activities of daily living, *QoL* quality of life, *KOOS-PF* KOOS patellofemoral subscale, *VAS* visual analogue scale

Square parentheses indicate possible score range, from best to worst possible score.

Number of observations: ^a^ *n* = 12; ^b^ *n* = 15; ^c^ *n* = 11; ^d^ *n* = 11.

## Discussion

Our findings indicate that a full-scale RCT of contoured foot orthoses versus flat insoles for adolescents with PFP would not be feasible with the protocol used in this study, based on our a priori criteria to inform feasibility. Although our observed recruitment rate (1.2 participants per week) exceeded the pre-specified criterion of 1 participant per week, parameters for minimum adherence with shoe insert wear and drop-out rate were not met. Of the 15 participants (42%) who provided adherence data, only 7 (47%) met minimum adherence of 2 h per day, 5 days per week for at least 7 out of 12 weeks. At 3 months, 28% of participants had dropped out (10/36), of which 4 were related to the COVID-19 pandemic. There were only minor, transient adverses event reported, and minimal co-intervention use. Participants perceived both interventions to be credible, and results of the Credibility and Expectancy Questionnaire demonstrated success of blinding. The method of patient-reported outcome collection was feasible and acceptable in this group.

Our recruitment strategy involved recruiting adolescent volunteers from the community via advertisements, as well as contacting adolescents with PFP from a pre-existing database. Combined, this strategy led to a recruitment rate of 1.2 participants per week over 30 weeks. However, we acknowledge that a future full-scale RCT may not have access to such a database, and thus is likely to rely solely on recruitment from the community. If the 6 participants who were recruited from the pre-existing database are not considered, our recruitment rate for the remaining 30 participants was 1 participant per week. This also meets our a priori  criterion [17]. A full-scale trial in this population may need to run across multiple sites or use a broader recruitment strategy (e.g., through schools) to ensure efficient recruitment of the target sample size. Of the advertising methods used, social media was most successful, accounting for 90% of participants recruited from advertising (27/30). Future studies recruiting adolescents with PFP should consider using social media as their primary advertising method.

Our observed percentage of total volunteers entering the study (12.9%) is in line with previous PFP RCTs. Rathleff et al. [13] reported a 16.7% inclusion rate of adolescents with knee pain at screening, who were recruited through schools. Collins et al. [11] included 11.7% of adult volunteers with PFP, who were recruited through community advertising. The most common reasons for excluding volunteers were that they were already wearing foot orthoses (*n* = 70), had sustained a previous lower limb injury (*n* = 29), or had anterior knee pain from another source (e.g. patellar tendinopathy, Osgood-Schlatter disease) (*n* = 18). Interestingly, 97 out of 260 (37%) adolescents who responded to advertisements and completed the initial online screening form did not respond to follow-up email or telephone communications. Therefore, it is not possible to know how many of these volunteers were potentially eligible for physical screening. Our observed rate of non-response is substantially higher than the proportion of adults with PFP who did not respond to follow-up communications after volunteering for a previous study (182/1530, 11.9%) [11]. Although the reasons for this are unclear, adolescents are likely to have different considerations and preferences for communicating or engaging with researchers and barriers to participation, which need to be explored further.

We evaluated two aspects of adherence – logbook completion and time wearing the allocated inserts. 42% (15/36) of participants returned their logbooks, suggesting that data from the logbooks may not be representative of the whole group. Because the logbooks were the primary source of data relating to insert wear time, this also suggests that our insert adherence data may not be representative. 63% (5/8) of participants in the contoured foot orthoses group reported wear time that met our predefined criteria for minimum adherence for more than half of the 3-month study period, compared to 29% (2/7) of participants from the flat insole group, although average wear time was similar for both groups (4.5 hours per day, or 32 hours per week). This is lower than a previous study in older adults with patellofemoral osteoarthritis (mean age 60 years), where logbooks were completed by 69% of participants and average insert wear time was 37 hours per week [35]. Our findings are likely to overestimate insert wear, as participants who completed and returned their logbooks may be more likely to be adherent with other aspects of the study, such as wearing their allocated insert. Further research is needed to determine the most effective methods of facilitating and monitoring adherence in this population. For example, app- or web-based methods may be more acceptable to adolescents with PFP due to the high use of electronic devices in this demographic [36]. Notwithstanding this, the low adherence rates that we observed may indicate that shoe inserts alone are not a viable intervention in this population. This requires further exploration to determine the barriers and facilitators to wearing shoe inserts, as well as footwear preferences of adolescents with PFP. For example, logbook data suggests that our participants spent a substantial amount of time wearing flip-flops or similar footwear, which coincided with days of low shoe insert wear time. Alternatively, there may be other factors that affected adherence with wearing the shoe inserts, such as the COVID-19 pandemic. Participants were in lockdown (confined to their homes) during the study period and were undertaking home schooling. This likely resulted in participants substantially reducing their shoe wear time, compared to normal school attendance and sport participation.

We set an a priori drop-out rate of ≤20% to inform feasibility [17]. Overall, 28% (10/36) of participants were lost to follow up at 3 months. 31% (11/36) of participants did not attend any physiotherapy appointments, and therefore did not receive their allocated intervention. It is important to explore the reasons why these adolescents dropped out or did not attend physiotherapy. One consideration is the impact of the COVID-19 pandemic. Four participants were due to schedule their initial physiotherapy appointments when COVID-19 restrictions were coming into effect in Queensland. If these participants are not considered, then the total loss to follow-up is 17% (6/36), which is within our pre-specified feasibility criterion, and the number of participants not attending any physiotherapy appointments is 7 (19%). However, it is likely that there were other barriers to our participants attending physiotherapy appointments, such as relying on parents for transport and the geographic location of the clinics involved in our trial. These need to be addressed if a full-scale RCT is to be feasible.

There were minimal differences between groups on the Credibility and Expectancy Questionnaire. At baseline, prior to randomization, median scores for the two groups were identical or within 1 response category. After receiving the allocated intervention, the group who received flat insoles demonstrated lower scores on all items of the Credibility and Expectancy Questionnaire, although median scores for the two groups were within 1.5 categories. The similarity in scores between groups after receiving the intervention, and between baseline and follow up for item 6 (*By the end of your therapy, how much improvement in your symptoms do you really* *feel* *will occur?*) indicates success of blinding. No other studies have assessed credibility and expectancy outcomes in adolescents with PFP. However, success of blinding was demonstrated in a study investigating lower limb injury risk in military recruits randomized to receive contoured prefabricated foot orthoses or flat insoles [37]. This suggests that flat insoles are an appropriate comparator for foot orthoses, including for adolescents with PFP.

Our findings also indicate that adolescents with PFP have minimal adverse events associated with wearing shoe inserts. Six participants reported rubbing, blisters and foot pain associated with both types of shoe inserts, most often in the first 2 weeks of wear. However, these were minor, typically resolved in 1-2 days, and did not preclude ongoing insert wear. Similar to previous studies involving shoe inserts for knee pain [11, 15, 38], our findings indicate that contoured foot orthoses, as well as flat shoe inserts, are tolerated well by adolescents with PFP.

There are two key limitations of this study. Firstly, there was a higher drop-out rate observed in the flat insole group at 3 months (42% in flat insole vs. 12% in foot orthoses group). Half of the participants lost to follow-up in the flat insole group were due to the COVID-19 pandemic (4/19, 21%), prior to receiving their allocated inserts. We observed similar outcomes on the Credibility and Expectancy Questionnaire after participants had received their allocated shoe inserts, suggesting that there may be other factors affecting retention in this group. Secondly, only 42% of participants returned the logbook, resulting in incomplete data for adherence with the shoe inserts, use of co-interventions and pain relief, activities undertaken, and adverse reactions. Thus, it is not clear whether these data are representative of the entire cohort.

## Conclusion

Based on a priori criteria for feasibility, our findings indicate that a full-scale RCT comparing contoured foot orthoses to flat insoles in adolescents with PFP would not be feasible using the current protocol, due to low rates of retention and adherence to the interventions. We recommend that feasibility issues are addressed prior to conducting a full-scale RCT, with protocol modifications to facilitate participant retention, logbook completion, and shoe insert wear.

## Data Availability

The datasets used and/or analysed during the current study are available from the corresponding author on reasonable request.

## Abbreviations

CONSORT: Consolidated Standards of Reporting Trials
EQ-5D: EuroQOL-5D
EVA: Ethylene-vinyl acetate
GROC: Global Rating of Change
HAPPi Kneecaps!: sHoe inserts for Adolescents with Patellofemoral PaIn
KOOS-Child: Knee injury and Osteoarthritis Outcome Score Child Version
KOOS-PF: Knee injury and Osteoarthritis Outcome Score patellofemoral subscale
PASS: Patient Acceptable Symptom State
PFP: Patellofemoral pain
RCT: Randomised controlled trial
REDCap: Research Electronic Data Capture
SPIRIT: Standard Protocol Items: Recommendations for Interventional Trials
